# Sex-specific effects in how childhood exposures to multiple ambient air pollutants affect white matter microstructure development across early adolescence

**DOI:** 10.21203/rs.3.rs-3213618/v1

**Published:** 2023-08-17

**Authors:** Megan Herting, Devyn Cotter, Hedyeh Ahmadi, Carlos Cardenas-Iniguez, Katherine Bottenhorn, W. James Gauderman, Rob McConnell, Kiros Berhane, Joel Schwartz, Daniel Hackman, Jiu-Chiuan Chen

**Affiliations:** University of Southern California; University of Southern California; University of Southern California; University of Southern California; University of Southern California; Keck School of Medicine of USC; University of Southern California; Columbia University; Harvard TH Chan School of Public Health; University of Southern California; USC

**Keywords:** air pollution, brain tract development, white matter microstructural integrity, longitudinal, adolescence, sex differences

## Abstract

Ambient air pollution is ubiquitous, yet questions remain as to how it might impact the developing brain. Large changes occur in the brain’s white matter (WM) microstructure across adolescence, with noticeable differences in WM integrity in male and female youth. Here we report sex-stratified effects of fine particulate matter (PM2.5), nitrogen dioxide (NO2), and ozone (O3) on longitudinal patterns of WM microstructure from 9–13 years-old in 8,182 (49% female) participants using restriction spectrum imaging. After adjusting for key sociodemographic factors, multi-pollutant, sex-stratified models showed that one-year annual exposure to PM2.5 and NO2 was associated with higher, while O3 was associated with lower, intracellular diffusion at age 9. All three pollutants also affected trajectories of WM maturation from 9–13 years-old, with some sex-specific differences in the number and anatomical locations of tracts showing altered trajectories of intracellular diffusion. Concentrations were well-below current U.S. standards, suggesting exposure to these criteria pollutants during adolescence may have long-term consequences on brain development.

## Introduction

Outdoor ambient air pollution is exceedingly being recognized for its consequential neurotoxicant effects.^1^ Criteria pollutants include, but are not limited to, particulate matter with diameter < 2.5 μm (PM_2.5_) and nitrogen dioxide (NO_2_), both of which result from combustion of gasoline, oil, diesel fuel, coal, or wood, as well as ground-level ozone (O_3_) that results from ultraviolet light-driven photooxidation of volatile organic compounds and other precursors. When inhaled deeply into the lungs, these pollutants cause an innate immune response at the level of the lung alveoli, leading to increased systemic inflammation^2^; inflammatory immune components in the bloodstream can then enter the brain via a compromised blood-brain barrier or by traveling along the vagus nerve, by-passing the blood-brain barrier.^3^ Children are thought to be especially vulnerable to the harmful effects of air pollution due to their comparatively higher respiratory rates, rate of neural change, and time spent outside compared to adults.^4,5^ Yet, there is uncertainty regarding the potential long-term effects of exposure on the dynamic neural processes that occur across adolescence and whether these effects vary between the sexes.^6,7^

The brain undergo remarkable changes during the second and third decades of life, with robust changes in white matter maturation.^8^ White matter comprises over half of the brain and these tracts are known as the information superhighways of the brain, connecting gray matter regions in the service of neural network organization. This integrated and efficient structural connectivity plays an important role in information processing, working memory, learning, and mental and emotional health outcomes.^9^ Support cells known as oligodendrocytes produce myelin, insulating the axons to form white matter tracts, but are known to be susceptible to damage resulting from inflammation. Given the known systemic and neuroinflammatory consequences of air pollution, white matter microstructure development may be particularly vulnerable to environmental neurotoxicant damage.^1^ Supporting this notion are recent ecological and cross-sectional diffusion imaging studies showing that exposure to air pollutants is associated with differences in white matter macro- and microstructure in youth. Calderón-Garcidueñas and colleagues^10^ found an increased incidence of white matter pathology in the prefrontal cortex in young people from Mexico City exposed to high levels of outdoor air pollution. Multiple studies using the Generation R cohort, a large birth cohort based in the Netherlands, found that prenatal exposure to PM_2.5_ and childhood exposure (0–4 years old) to PM_2.5_, NO_2_, and nitrogen oxides (NOx) were associated with lower global fractional anisotropy, suggesting reduced white matter microstructural integrity, when measured at ages 9–12 years.^11,12^ More recently, Peterson and colleagues^13^ assessed the impact of prenatal exposure to PM_2.5_ on neurodevelopment in 6–14 year-olds across multiple imaging modalities with findings that indicate that exposure to higher PM_2.5_ was associated with higher average diffusion coefficient in white matter fiber bundles, indicating less myelin, reduced fiber density, and/or less directional fiber coherence. Taken together, these initial studies suggest that ambient air pollution exposure during development is linked to differences in white matter microstructure. The noted discrepancies in directionality might stem from divergent study samples, differences in the timing of exposure and/or age of brain assessment, and/or differences in MRI techniques; thus, additional studies are warranted to more fully understand how air pollution exposure influences white matter maturation across childhood and adolescence.

It is plausible that air pollution effects on white matter microstructure, especially during development, may differ based on biological sex. Sex has largely been implicated by both epidemiological and experimental animal studies in how air pollution affects health outcomes,^14^ and there are well-established sex differences in white matter development.^15,16^ Despite this, it remains to be determined if sex-specific effects exist in the susceptibility of white matter maturation to the putative effects of air pollution. For example, while notable sex differences have been observed in various associations between early life air pollution exposure and neurobehavioral and cognitive outcomes,^17,18^ white matter neuroimaging studies to date have either not examined potential sex differences,^12,19^ or failed to find effects.^11,13^ In addition, all MRI studies to date, while they may have examined different developmental windows of pollutant exposure, have been based on a single MRI assessment. Thus, while providing important information about the potential harmful effects of air pollution on white matter integrity, these studies have been limited by their cross-sectional design and by overlooking potentially important sex-specific relationships, restricting their ability to comment on how air pollution might impact key neurodevelopmental trajectories in male and female youth. To more fully characterize air pollution exposure as it relates to white matter brain maturation, longitudinal studies considering sex-specific effects are required.

Moving forward, new advancements in biophysical modeling of diffusion imaging data, such as restriction spectrum imaging (RSI), holds great promise in furthering our understanding as to how air pollution impacts white matter development. By quantifying restricted normalized isotropic (RNI) and directional (RND) diffusion, RSI can be used to infer the biological processes contributing to white matter microstructure development, such as diffusion within support cells as well as axon or fiber bundles, respectively.^20,21^ As such, our group recently leveraged RSI to examine how ambient PM_2.5_ exposure was cross-sectionally associated with patterns of white matter RNI and RND in children aged 9–10 years. We found that greater PM_2.5_ exposure was linked to increased RNI, which may indicate swelling or activation of support cells, potentially as a consequence of inflammatory processes near the affected tracts.^22^ Given both the cross-sectional nature of our initial study as well as recently reported changes in RNI and RND with age,^21^ questions remain as to whether ambient air pollution impacts white matter microstructural development. Moreover, no study to date has examined how gaseous criterion pollutants may impact these novel metrics of white matter health.

In this longitudinal study, we aimed to determine if exposure to PM_2.5_, NO_2_, and O_3_, at ages 9–10 years has long-term effects on trajectories of white matter microstructure development over a 2-year follow-up period from late childhood into early adolescence. For a more detailed evaluation of the potential mechanisms by which air pollution exposure may affect various neural processes underlying pediatric white matter development, we quantified white matter microstructural integrity using RSI to isolate intracellular spaces. Given sex-specific effects in environmental neurotoxicity^23^ as well as in white matter microstructural development as measured with RSI,^15^ we examined air pollution effects in each sex separately as opposed to including a pollutant-by-sex interaction term, reducing the potential for bias.^24^ We hypothesized that exposure to higher concentrations of outdoor air pollution during late childhood would be associated with altered trajectories of white matter microstructural development during the transition to early adolescence and that regions affected may be sex-specific. Lastly, air pollution exposure concentrations within the ABCD Study fall well below the U.S. EPA’s National Ambient Air Quality Standards (NAAQS),^25,26^ allowing the current study to examine potential adverse neurodevelopmental effects from exposures that adhere to or fall below current environmental regulations. This is especially important given that despite improved air quality, adverse brain health effects continue to be detected at low levels of exposure.^22, 25–27^ Thus, the current study may provide actionable information to policy makers actively working to update risk assessment of air pollution exposure on human health.

## Results

**Figure 1:**
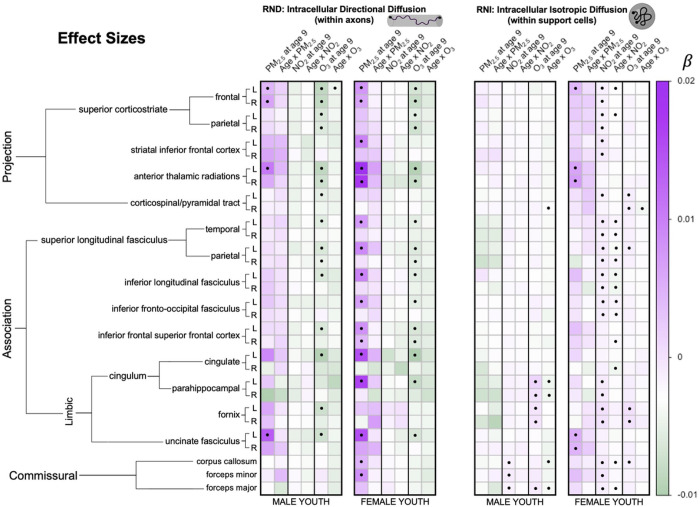
Heatmap demonstrating the direction and magnitude of effect size for each association between coefficients of interest (fixed effects of each pollutant as well as age-by-pollutant interactions) and white matter microstructure quantified by restricted directional (RND) and isotropic (RNI) fractions per white matter tract, after adjusting for all socioeconomic, demographic, and MRI precision variables, as well as other pollutants. Significant models that passed FDR-correction (>0.05) are denoted with a closed circle [●].

**Figure 2:**
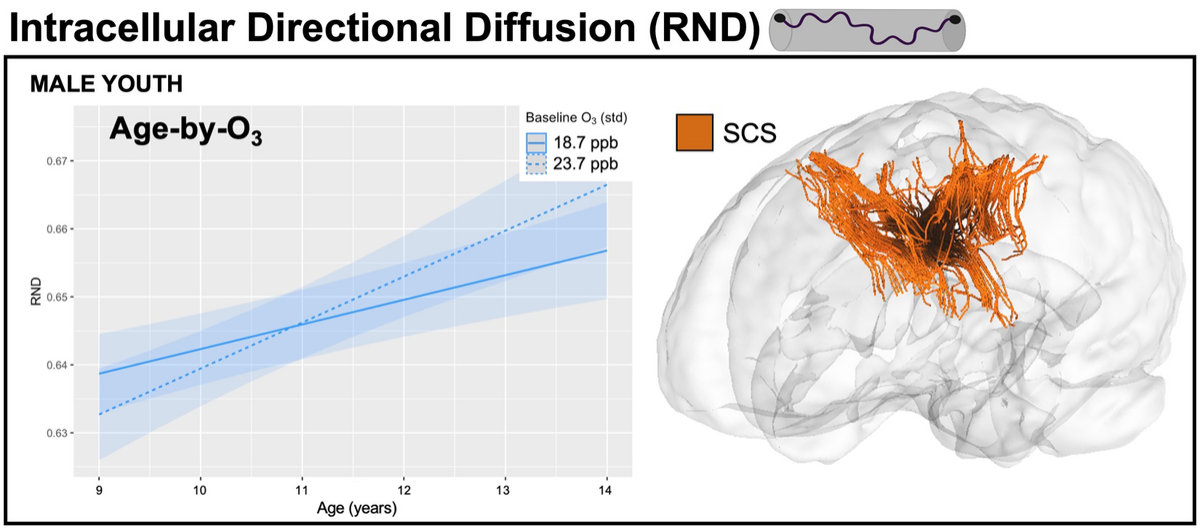
Significant (FDR-p < 0.05) effects of age-by-O_3_ interaction on the left frontal superior corticostriate intracellular directional diffusion (RND) in male youth only after adjusting for all covariates, representing the effect of O_3_ on change of this tract over time. On the age-by-pollutant interaction plot, the solid line represents the mean value of pollutant within our sample and the dashed line represents 5 units higher than the mean.

**Figure 3:**
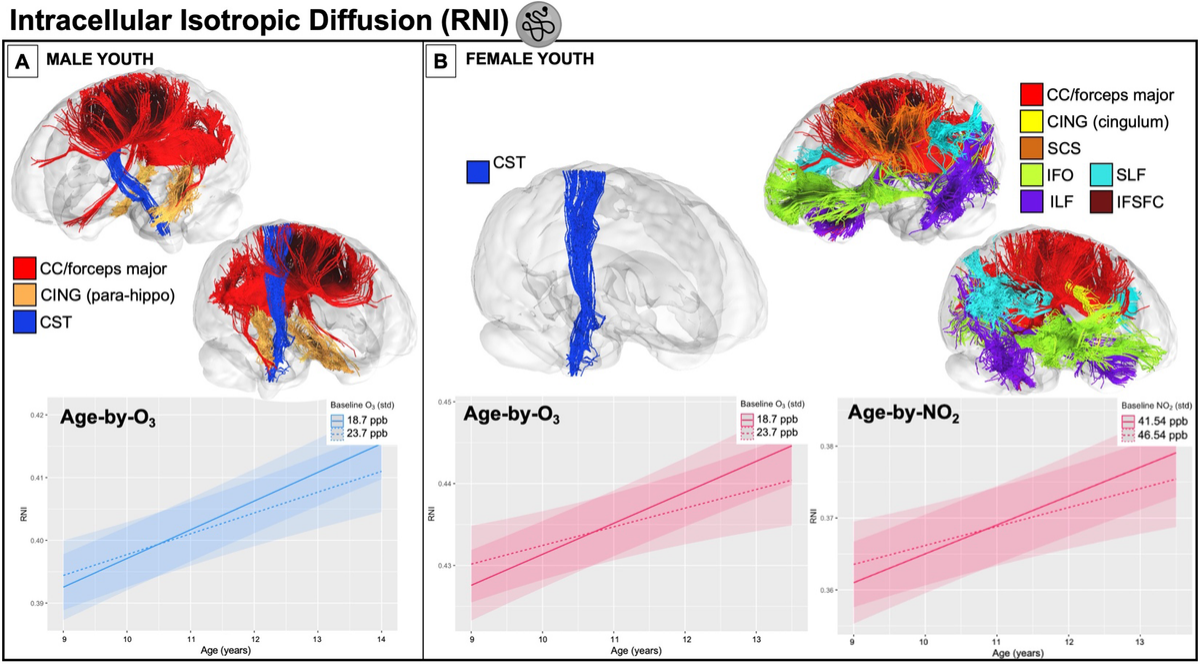
A) Significant (FDR-p < 0.05) effects of O_3_ on changes in intracellular isotropic (support cells) diffusion (RNI) of the corpus callosum, forceps major, bilateral parahippocampal cingulum, and the right corticospinal tract in male youth only after adjusting for all covariates; the plot is representative as all associations were of a similar magnitude and significance level. B [left]) Significant (FDR-p < 0.05) effects of O_3_ on changes in the right corticospinal tract RNI in female youth only. B [right]) Significant (FDR-p < 0.05) effects of NO2 on changes on changes in near-global RNI in female youth only after adjusting for all covariates; the plot is representative as all associations were of a similar magnitude and significance level. Results are driven by the left superior corticostriate (frontal and parietal), right cingulate cingulum and inferior frontal superior frontal cortex, as well as bilateral inferior longitudinal fasciculi, inferior fronto-occipital fasciculi, superior longitudinal fasciculi (parietal and temporal), forceps major, and corpus callosum. On all age-by-pollutant interaction plots, the solid line represents the mean value of pollutant within our sample and the dashed line represents 5 units higher than the mean.

8,182 participants from the ABCD Study, consisting of 21 major urban areas across the U.S., were analyzed to investigate how one-year of annual exposure influences attained white matter microstructure at age 9 as well as changes in white matter microstructure trajectories from 9–13 years of age in female and male youth separately. Specifically, we implemented a multi-level modeling approach to first examine age-only effects and then conducted sex-stratified multi-pollutant models to examine how each exposure influenced RNI and RND at age 9 (i.e., main pollutant effect) as well as modified trajectories of development (i.e., an age-by-pollutant interaction). An ensemble-based model approach was used to assign a one-year annual average PM_2.5_, NO_2_, and O_3_ concentration to the primary address for each child at study enrollment (corresponding to their enrollment age of 9–10 years). Pollutant concentrations (PM_2.5_ = 7.69 ug/m^3^; NO_2_ = 41.5 ppb; O_3_ = 18.7 ppb) were significantly lower than the current EPA standards (one sample t-tests against EPA standards: PM_2.5_: t = −304.27, NO_2_: t = −659.96, O_3_ (8-hr): t = −697.26, all p’s < 2.2e-16). Development of white matter tracts was assessed using RNI and RND, which reflects intracellular water in support cells and intra-axonal diffusion, respectively. Moreover, we adjusted for key confounders, including race/ethnicity, household income, highest parental education, urbanicity, handedness, season of MRI scan, scanner manufacturer, tract volume, head motion, and the two pollutants not included in the pollutant-by-age interaction term. To begin, we first replicated previous white matter development findings from Palmer and colleagues^21^ showing both RNI and RND increased over time from ages 9–13 years (Supplemental Table 2). Next, exposure to PM_2.5_, NO_2_, and O_3_ was found to be associated with differences in intracellular WM microstructure at age 9, whereas only NO_2_ and O_3_ significantly moderated changes in white matter development from 9 to 13 years of age (as seen by significant pollutant-by-age interactions). Moreover, while directionality of effects was similar in both male and female youth, some sex specific effects were seen in both the number and anatomical locations of tracts affected by pollutant. For RND, PM_2.5_ had more widespread effects in female youth, whereas O_3_ affected both sexes similarly, and NO_2_ had no effects across either sex. For RNI, PM_2.5_ and NO_2_ affected more tracts in female youth with little or no effect in male youth, while O_3_ effects were more prevalent in limbic association tracts in male youth and included the corticospinal tract in female youth. All results can be found in Figs. 1–3 and Supplemental Tables 3–5.

### White Matter Microstructure Development from 9–13 years-old

Global RNI and RND increased over time from ages 9–13 years (Supplemental Table 2). There were almost no significant sex differences in the longitudinal change over RNI or RND over time (age-by-sex interactions FDR p’s > 0.05), except for the forceps minor. In this tract alone, RNI in male youth increased faster than female youth over time; RND increased in male youth but did not change in female youth over time (Supplemental Fig. 2). We did observe a significant relationship between sex and near-global RNI and RND at age 9, in that male youth had lower attained RNI and RND in almost all tracts tested (Supplemental Table 2).

### Effects of PM_2.5_ on White Matter Microstructure Development

Higher PM_2.5_ exposure was associated with higher RND at age 9 in the bilateral frontal superior corticostriate, left anterior thalamic radiation, and left uncinate fasciculi in both sexes. In addition, for female youth higher PM_2.5_ exposure was also associated with higher RND at age 9 in the left striatal inferior frontal cortex, right anterior thalamic radiation, and left superior longitudinal fasciculi (temporal and parietal), left inferior longitudinal fasciculus, left inferior fronto-occipital fasciculus, bilateral inferior to superior frontal cortex, left cingulum (cingulate and parahippocampal), and corpus callosum (driven by the forceps minor). There were no significant age-by-PM_2.5_ interactions on RND (FDR p’s > 0.05), suggesting that PM_2.5_ exposure does not modify RND development between the ages of 9–13 years of age.

In female youth, exposure to higher PM_2.5_ concentrations were associated with higher RNI at age 9 in the left frontal superior corticostriate as well as in the bilateral anterior thalamic radiations and uncinate fasciculi. PM_2.5_ exposure did not significantly relate to RNI at age 9 in male youth and no significant PM_2.5_-by-age interactions were seen in either sex (FDR p’s > 0.05), suggesting that PM_2.5_ exposure does not modify RNI development between the ages of 9–13 years of age.

### Effects of NO_2_ on White Matter Microstructure Development

There were no significant effects of NO_2_ exposure on RND for either sex at age 9 or on RND development over time (i.e., PM_2.5_-by-age interactions FDR p’s > 0.05).

For RNI, NO_2_ exposure was associated with higher RNI in the corpus callosum, including at both the forceps major and minor, at age 9 in both sexes. In female youth, additional tracts showed this significant positive association between NO_2_ exposure and RNI at age 9, including bilateral frontal and parietal superior corticostriate, bilateral striatal inferior frontal cortex, left corticospinal tract, bilateral temporal and parietal superior longitudinal fasciculi, bilateral inferior longitudinal fasciculi, bilateral inferior fronto-occipital fasciculi, bilateral cingulum (parahippocampal portion), and bilateral fornix. NO_2_ exposure also significantly influenced RNI changes with development from 9–13 years in female youth in a number of tracts (i.e., NO_2_-by-age interactions FDR p’s < 0.05), including the left frontal and parietal superior corticostriate, bilateral temporal and parietal superior longitudinal fasciculi, bilateral inferior longitudinal fasciculi, bilateral inferior fronto-occipital fasciculi, left cingulate and right inferior frontal superior frontal cortex, and bilateral corpus callosum (driven by the forceps major)(Fig. 3). In the majority of these white matter tracts showing both the main effects of NO_2_ at age 9 and NO_2_-by-age in female youth, higher NO_2_ exposure was related to higher levels of RNI at age 9, but with reduced increases in RNI with age (i.e., smaller positive slope over time). However, in the left cingulate and right inferior frontal superior frontal cortex RNI was similar at age 9, but with reduced increases in RNI with age (i.e., smaller positive slope over time). In males, no significant NO_2_-by-age interactions were found.

### O_3_ Effects on White Matter Microstructure Development

Exposure to higher concentrations of O_3_ were associated with lower RND at age 9 in both sexes in the bilateral frontal and parietal superior corticostriate, bilateral anterior thalamic radiations, left temporal superior longitudinal fasciculus, bilateral parietal superior longitudinal fasciculi, left inferior longitudinal fasciculus, left inferior to superior frontal fasciculus, left cingulate, and left uncinate fasciculus. Additional tracts showing a negative association between O_3_ and RND at age 9 included the left corticospinal tract and left fornix in male youth, but the left inferior fronto-occipital fasciculus and left parahippocampal region of the cingulum in female youth. Beyond O_3_ effects at age 9, a significant O_3_-by-age interaction was seen for the left frontal superior corticostriate in male youth, with higher O_3_ concentrations related to greater increases in RND with age over time (Fig. 2). No significant O_3_-by-age interactions were found in female youth.

Exposure to higher concentrations of O_3_ were associated with higher RNI at age 9 in bilateral fornix in both sexes. In addition, higher concentrations of O_3_ were associated with higher RNI at age 9 in bilateral parahippocampal region of the cingulum, forceps major of the corpus callosum in male youth, but bilateral corticospinal tract, left parietal superior longitudinal fasciculus, and corpus callosum in female youth. A significant O_3_-by-age interaction was seen in the right corticospinal tract in female youth, with higher O_3_ exposure linked to higher RNI levels at age 9, but reduced increases in RNI with age (Fig. 3). In males, O_3_-by-age interactions were seen for the right corticospinal tract, bilateral parahippocampal portion of the cingulum, and corpus callosum including the forceps major (Fig. 3), with higher O_3_ exposure linked to reduced increases in RNI with age from 9–13 years.

## Discussion

This is the first longitudinal, nationwide study in the U.S. to demonstrate that outdoor air pollution is linked to altered white matter microstructure development in today’s youth, with differential patterns of tracts affected in male and female youth. We find that exposure to even relatively low levels of pollutants is associated with disruptions in white matter at age 9 as well as developmental trajectories from ages 9–13 years in male and female youth. Criteria pollutants were significantly associated with changes in both intracellular isotropic diffusion, which may reflect changes in number, activation, or damage to glial support cells, such as microglia, astrocytes, or oligodendrocytes,^21^ as well as intracellular directional diffusion, which is thought to index changes in axonal caliber, density, and/or myelination.^21^ Affected tracts include projection, association, and commissural fibers that connect networks of brain regions important in planning and execution of complex and goal-oriented behaviors.^28^ Most importantly, the notable effects of ambient air pollution on white matter development were seen at concentrations that fall well-below current EPA standards. These findings lend further support towards a growing body of literature and the recent recommendations from the World Health Organization^29^ that suggests that air quality standards should be lowered to protect brain health of developing youth.

### Low-level Air Pollution and Pace of White Matter Brain Development

Previous studies have identified robust developmental changes in white matter.^8^ Underlying microstructural changes include increases in both restricted and isotropic intracellular diffusion from ages 9–14 years,^21^ representing increases in axonal density and/or caliber, myelination, and number or size of support cells. White matter development during the second and third decade of life is intrinsically linked with the brain’s ability to efficiently support cognitive, behavioral, and emotional functioning in everyday life.^30,31^ Moreover, a wide body of literature suggests the pace of brain development, for one’s chronological age, is important. Specifically, steady and prolonged neuroplasticity is considered essential, as both delayed and accelerated brain maturation have been linked with impaired cognitive and emotional development.^31–34^ Factors that accelerate the pace of brain development may especially be harmful in that it might not ensure sufficient time to learn from and adapt to everyday experiences during adolescence.^35,36^ The current findings suggest that exposure to pollutants during childhood may lead to a more advanced (i.e., “older”) phenotype, indicated by higher intracellular directional (associated with PM_2.5_) and isotropic (associated with all three pollutants with NO_2_ affecting the most tracts) diffusion in white matter tracts important for executive function and emotional regulation at age 9 as well as in slower increases (associated with NO_2_ and O_3_) in intracellular isotropic diffusion in these tracts over time as children transition to adolescence. On the other hand, O_3_ is coupled with a more immature (i.e., “younger”) white matter microstructure phenotype, indicated by lower intracellular directional diffusion at age 9, followed by an accelerated pace of maturation seen over time. As the first study to relate air quality to longitudinal changes in white matter microstructure, our findings reiterate the potential harms of even low-levels of air pollution on-par with what is considered acceptable by the EPA, on trajectories of white matter development. While air quality information is only currently available for one-year in the ABCD Consortium, future studies are needed to examine potential cumulative effects and to identify any periods of greater vulnerability during development, like those found in the Generation R cohort of children from the Netherlands.^11,12,19^ Nonetheless, given the associations between air quality over a single one-year period and white matter development, it is reasonable to expect the influence of longer-term exposure across the second and third decade of life may have escalating implications.

### Pollution Effects on White Matter Development – Potential Diverse Mechanisms of Neurotoxicity

The current study uses parameters from a multi-compartment modeling technique that, unlike conventional DTI metrics, is thought to better quantify spherical or elongated shapes of intracellular diffusion in intra- and extracellular tissue.^20^ This allows for a more detailed characterization of air pollution’s neurotoxic effects on white matter development, as biophysical models of spherical and directional intracellular diffusion mirror biological microstructure such as support cells and myelin, respectively.^20^ Animal exposure studies suggest pollution causes both neuroinflammation as well as damage to myelin sheaths.^37–39^ PM exposure has been associated with cell death, neuroinflammation, oxidative stress, damage to neurovascular units and endothelial cells, and weakening of tissue barriers across organ systems (e.g., nasal, lung, gastrointestinal, and blood-brain barriers).^40,41^ This neuroinflammatory cascade plausibly results from the infiltration of the brain by either ultrafine particles, PM-adsorbed soluble metals, and/or immune cells.^42^ Immune cells, once inside the brain after passing through a compromised blood-brain barrier, can attack myelin sheaths resulting in microstructural damage to white matter tracts.^43^ Other potentially damaging pathways include PM-associated increases in misfolded protein aggregates, which can be toxic, and dysfunction in their lysosomal-mediated degradation, even in children and young adults.^44^ NO_2_ may impact the brain through similar mechanisms, by inducing oxidative stress and subsequent apoptosis,^45^ as well as mitochondrial dysfunction.^46^ Mitochondrial dysfunction is a potentially important factor in how NO_2_ affects white matter because it has been associated with the degeneration of oligodendrocytes.^47,48^ We find that exposure to higher PM_2.5_ and NO_2_ at age 9 is associated with higher restricted directional and isotropic diffusion respectively in female youth, implying that myelination is increasing sooner than expected. However, over time this pollutant-associated precocious development leads to sharper declines in microstructural integrity, such that the increase in restricted diffusion (both isotropic and directional) is either slowed or reversed, potentially representing myelin and support-cell damage. In contrast, exposure to higher O_3_ at age 9 is associated with lower restricted directional diffusion and this seemingly delayed development leads to exaggerated increases in microstructural integrity over time, potentially indicating hypermyelination. Unlike PM_2.5_ and NO_2_, O_3_ is not able to penetrate the lung and enter the brain via systemic circulation. Instead, it can cause an innate immune reaction at the level of the lung alveoli and subsequent upregulation of circulating inflammatory cytokines, implying that its neurotoxic effects are largely caused by increased systemic inflammation.^49^ Generally, pollutants are thought to infiltrate brain tissue by traveling along the olfactory nerve to the olfactory bulb, resulting in astrocyte and microglial activation and the resulting neuroinflammatory cascade that damages the brain’s white matter.^50^ Moreover, consistent with the idea that air pollution may be a physical stressor that disrupts white matter development, both O_3_ and PM_2.5_ pollution exposure can activate the hypothalamo-pituitary-adrenal (HPA) axis, triggering cortisol release and glucocorticoid upregulation, while altering glucocorticoid-regulated gene expression to increase glucocorticoid activity within the brain itself.^51^ Congruent with these findings, changes in morning cortisol serum levels were found in adolescents as a result of exposure to O_3_ and PM_2.5_ in the months prior to assessment.^52^ However, while O_3_ exposure was associated with increases in morning cortisol levels, PM_2.5_ was associated with decreases. Studies have found that glucocorticoids can both inhibit and promote the proliferation of oligodendrocyte progenitors depending on dose, duration, and location.^51,53^ In light of this and our similarly opposing effects of PM_2.5_ and O_3_, air pollutants may act through distinct mechanistic pathways that lead to unique and even opposite patterns of endocrine dysregulation, together with their combined effects on neuroinflammatory pathways. It is also important to note that while PM_2.5_ effects were more focal compared to more widespread changes observed with NO_2_ and O_3_, the PM_2.5_ effect sizes were on an order of magnitude larger than those of the gasses. This is consistent with prior literature citing PM_2.5_ as one of the most harmful pollutants to human health,^54^ even at the relatively low doses we observe in the ABCD cohort.

### Sex Differences in Vulnerabilities of Air Pollution on White Matter Development

Given there are well-documented sex differences in the rate of white matter development as well as in air pollution exposure and inclusion of a sex-by-pollutant interaction could introduce bias,^24^ the current study examined potentially harmful effects of air pollution exposure on white matter development in each sex separately. Although previous cross-sectional air pollution and white matter studies did not find sex-specific effects,^11,13^ the current sex-stratified longitudinal findings highlight that while the directionality of air pollution effects were similar across both sexes, the tracts affected were sometimes sex-specific. As such, our results demonstrate that criteria pollutants influence brain white matter microstructure, albeit with PM_2.5_ and NO_2_ effects more prevalent in female youth and tracts affected by O_3_ differing between male and female youth. Systemic inflammation may be the main culprit responsible for observed noxious gas-associated findings (as discussed above), but with differential cellular and epigenetic vulnerabilities in male compared to female children. For example, there is some evidence that NO_2_ preferentially affects females – increases in NO_2_ have been associated with elevated serum protein gene product 9.5 (PGP9.5) in women, but decreased in men.^55^ Because PGP9.5 originates in the brain, elevated serum PGP9.5 may indicate a compromised blood-brain barrier, implying that increased exposure to ambient NO_2_ and subsequent oxidative stress may compromise the permeability of the blood-brain barrier. Females also may be at an increased risk of functional impairment from NO_2_. A recent study observed greater cognitive decline in women associated with increased exposure to NO_2_.^56^ However, other animal model studies indicate that females are more likely to be protected from air pollution’s negative effects by paraoxonase 2 (PON2), an enzyme with antioxidant and anti-inflammatory properties that is more highly expressed in the brains of females than males because it is modulated by estradiol^57,58^; or through NO_2_-mediated increases in prolactin gene expression, known for its anti-inflammatory properties in female but not male mice.^59^ Of course, many of these studies focus on women, with adult levels of estradiol; which may not directly translate to other stages of the life course, such as childhood and early adolescence, when most children are in the early stages of puberty, exhibiting much lower estradiol levels.^60^ Thus, sex differences in air pollution’s impacts on other organ systems may complicate sex differences in air pollution neurotoxicity. For example, known coupling of immune and endocrine systems^61^ indicates further research is necessary to understand differential effects of air pollution on pituitary-gonadal-adrenal function and/or sex steroid levels across various periods of the lifespan, which may contribute to these notable sex differences in white matter maturation.^15,62,63^ Similarly, additional research is warranted to understand the degree to which air pollution may alter epigenetics involved in the upregulation of myelin genes in males compared to females. Emerging areas of study suggest it is feasible that O_3_ might affect myelin-related epigenetic pathways located in the microbiome and act along the gut-brain axis to induce changes in brain structure, specifically in males. That is, hypermyelination of the prefrontal cortex has been observed in male (but not female) mice with altered gut microbiota,^64^ and associations between ozone-dependent microbiome changes and airway hyperresponsiveness are also only seen in male mice.^65^ Interestingly, prenatal pollutant exposure has also been linked to increased incidence of autism spectrum disorder (ASD) in male youth^17^ – a neurodevelopmental disorder with notable phenotypes of both regional hypermyelination^66^ and gut microbiome alterations.^67^ Taken together, additional animal and human-based gene-by-environment studies are necessary to further identify potential endocrine systems and myelin-related epigenetic pathways that may contribute to differential patterns of susceptibility to white matter maturation in developing male and female brains.

In conclusion, our results reveal important associations between criteria pollutants and white matter microstructure developmental trajectories during the transition from childhood to adolescence. With additional context from animal and human studies, we speculate that systemic and neuro-inflammatory processes may underlie pollutants’ effects on white matter health during this vulnerable neurodevelopmental period. Notably, the sex-stratified white matter changes identified here were observed at low levels of exposure, as exposure concentrations of the criteria pollutants examined fell well below current EPA standards, albeit they still exceed the latest WHO guidelines released in September 2021. In light of this, the current findings in this U.S.-based sample should be considered by the EPA when revising air pollution regulatory standards.

## Methods

### Study Population

Longitudinal data were collected as a part of the ongoing longitudinal ABCD Study, which enrolled 11,876 children at ages 9–10 years across 21 study sites. Study enrollment criteria included age (≤ 10-years-old at initial visit) and English language proficiency. Exclusion criteria included major medical or neurological conditions, history of traumatic brain injury, diagnosis of schizophrenia, moderate/severe autism spectrum disorder, intellectual disability, alcohol/substance use disorder, premature birth (gestational age < 28 weeks), low birthweight (< 1200 g), and contraindications to MRI scanning.^68^ All study procedures were approved by the centralized institutional review board at the University of California San Diego; each study site also obtained approval from their institutional review boards. Participants provided written assent and legal guardians provided written consent.

We used a subset of data from the ABCD Study, including magnetic resonance imaging (MRI) from the baseline and/or year-2 follow-up study visits and measures of participants’ age, sex at birth, and sociodemographics held constant from the baseline assessment. Only high-quality imaging scans completed before March 1, 2020 were included to remove potential confounding effects of stress inherent to the COVID-19 pandemic. We filtered for valid air pollution estimates (see quality control details below), and randomly selected one subject per family to reduce the number of hierarchical levels, uneven by study design (i.e., the number of both siblings and twins vary by site). Our final sample included 8,182 subjects across 21 study sites. Of these, 3,679 (45%) had two time points of high-quality DWI data, while 4503 (55%) had one DWI time point, either from the baseline or 2-year follow-up visit (see details below; Table 1). All data used here were obtained from ABCD’s 4.0 data release (http://dx.doi.org/10.15154/1523041).

### Ambient Air Pollution Estimates

Annual ambient air pollution concentration for PM_2.5_, NO_2_, and O_3_ were assigned to primary residential addresses of each child as previously described.^69^ Briefly, daily estimates were derived at a 1-km^2^ resolution using hybrid spatiotemporal models, utilizing satellite-based aerosol optical depth models, land-use regression, and chemical transport models,^69–71^ and averaged over the 2016 calendar year, corresponding with enrollment for the baseline assessment. One-year annual average concentrations were then assigned to primary residential address at the baseline assessment when children were aged 9–10 years. PM_2.5_ was positively correlated with NO_2_ (r = 0.21, p = 3.44e-81) and negatively correlated with O_3_ (r = −0.19, p = 1.78e-64); there was no correlation between NO_2_ and O_3_ (r = −0.02, p = 0.12).

### Diffusion Weighted Imaging (DWI): Acquisition, Processing, and Quality Control

A harmonized neuroimaging protocol was utilized across sites, given the differences in scanner manufacturer (3T Siemens, Phillips, or GE). The multi-shell DWI acquisition included a voxel size of 1.7 mm isotropic, implemented multiband EPI^72,73^ with slice acceleration factor 3, and included a fieldmap scan for B0 distortion correction. Seven b = 0 frames and 96 total diffusion directions at 4 b-values (6 with b = 500 s/mm^2^, 15 with b = 1000 s/mm^2^, 15 with b = 2000 s/mm^2^, and 60 with b = 3000 s/mm^2^) were collected.^74^ All images underwent distortion, bias field, and motion correction, and manual and automated quality control.^74^ After preprocessing, white matter tracts were identified using the probabilistic atlas AtlasTrack.^75^ Only images without clinically significant incidental findings (*mrif_score* = 1 or 2) that passed all ABCD quality-control parameters (*imgincl_dmri_include* = 1) were included in analysis.

### Restriction Spectrum Imaging (RSI)

Restriction spectrum imaging (RSI) utilizes all 96 directions in ABCD’s multi-shell acquisition protocol.^76^ RSI provides detailed information regarding both the extracellular and intracellular compartments of tissue within the brain.^20^ RSI model outputs are normalized measures, unitless on a scale of 0–1. We focused on restricted (intracellular) normalized isotropic signal fraction (RNI) and restricted normalized directional signal fraction (RND) white matter fiber tract ROIs created with AtlasTrack.^75^ We explored all tracts excluding summary tracts (14 in the left hemisphere, 14 in the right hemisphere, and 3 spanning both hemispheres), including the right and left fornix, cingulate cingulum, parahippocampal cingulum, corticospinal tract, anterior thalamic radiations, uncinate fasciculi, inferior longitudinal fasciculi, inferior fronto-occipital fasciculi, temporal and parietal superior longitudinal fasciculi, frontal and parietal superior corticostriate, striatal to inferior frontal cortex, and inferior frontal to superior frontal cortex, as well as the forceps major, minor, and corpus callosum.

### Predictors

Predictors were chosen using a directed acyclic graph and included demographic and socioeconomic variables: race/ethnicity (*race_ethnicity* variable with the following categories: *White, Black, Hispanic, Asian*, or *Other*), annual household income (USD; *>100K, 50–100K, < 50K*, or *Don’t Know/Refuse to Answer*), and highest household education (*Post-Graduate, Bachelor, Some College, High School Diploma/GED*, or *< High School Diploma*). Pollution levels are higher in minority communities and those from disadvantaged social status backgrounds due to structural racism and class bias increasing the likely proximity of these communities to major sources of pollution in the U.S. ^77,78^ Census Tract Urban Classification (*Rural, Urban Clusters*, or *Urbanized Area*) was included as air pollution levels vary by degree of urbanicity. We also included the subject-specific precision variable, handedness (*right, left*, or *mixed*) and MRI-related precision variables such as scanner manufacturer (*Siemens, Philips, GE*) to account for differences in both scanner hardware and software, average frame displacement (*mm*) to account for head motion, and tract volume. Lastly, due to the potential acute differences in seasonality of pollutant concentrations at the time of each visit, we included the meteorological season of the MRI scan date as an additional time-varying variable.

### Statistical Analyses

We used hierarchical linear mixed-effect models, as implemented in *lme4*::*lmer()* in R statistical software (Version 4.1.2.).^79^ We first tested a developmental model, examining the main effects of age and sex, as well as an age-by-sex interaction term. Next, we tested the longitudinal change of RSI outcomes in the context of specific pollutants (PM_2.5_, O_3_, and NO_2_), opting for sex-stratified models over including pollutant-by-age-by-sex or pollutant-by-sex interaction terms; the outcomes and a number of predictors in the model have demonstrated sex-specific effects and the inclusion of an interaction term would likely introduce bias.^24^ In each model, subjects nested within ABCD sites were modeled as random effects, to account for the multi-level data structure. Age was centered on the lowest age within our sample (107 months), resulting in a scaled age score of 0 equivalent to 8.9 years. We accounted for non-linearity of age by utilizing a piecewise linear spline model, placing a knot at median age of 127 months. This two-piece linear spline model was parameterized to include an overall effect of age and an age-deviation (ageD) term. To assess how air pollution exposures affect age-related maturation in WM microstructure, we have included an interaction of pollutants with the age-specific spline terms. Specifically, each model included the pollutant of interest (PM_2.5_, O_3_, or NO_2_), age, ageD, interactions of the specific pollutant with age and ageD, and all predictors discussed above. To account for co-exposure of the three criteria pollutants, we additionally controlled for the other two pollutants not included in the age-by-pollutant interaction term of interest. For example, we included NO_2_ and O_3_ as covariates in the model testing the effects of PM_2.5_, age, and age-by-PM_2.5_ (plus ageD, ageD-by-PM_2.5_, and previously mentioned predictors) on RSI outcomes. Parameters of interest included the fixed effects of the pollutant on attainted WM microstructure at age 9 (i.e., scaled age score of 0), age (i.e., time), and the age-by-pollutant interaction term to investigate how WM maturation may be altered by air pollution exposure. To account for multiple comparisons due to modeling RNI and RND for all available white matter tracts, we performed a false discovery rate (FDR) correction for 62 tests (31 total white matter tracts across both sexes).
